# Road traffic crash circumstances and consequences among young unlicensed drivers: A Swedish cohort study on socioeconomic disparities

**DOI:** 10.1186/1471-2458-10-14

**Published:** 2010-01-14

**Authors:** Christina L Hanna, Marie Hasselberg, Lucie Laflamme, Jette Möller

**Affiliations:** 1 Karolinska Institutet, Department of Public Health Sciences, Division of Global Health (IHCAR) Stockholm, Sweden; 2 Karolinska Institutet, Department of Public Health Sciences, Division of Public Health Epidemiology, Stockholm, Sweden

## Abstract

**Background:**

Young car drivers run a higher risk of road traffic crash and injury not only because of their lack of experience but also because of their young age and their greater propensity for adopting unsafe driving practices. Also, low family socioeconomic position increases the risk of crash and of severe crash in particular. Whether this holds true for young unlicensed drivers as well is not known. Increasing attention is being drawn to the prevalence and practice of unlicensed driving among young people as an important contributor to road traffic fatalities.

**Methods:**

This is a population-based cohort study linking Swedish national register data for a cohort of 1 616 621 individuals born between 1977 and 1991. Crash circumstances for first-time road traffic crash (RTC) were compared considering licensed and unlicensed drivers. The socioeconomic distribution of injury was assessed considering household socioeconomic position, social welfare benefits, and level of urbanicity of the living area. The main outcome measure is relative risk of RTC.

**Results:**

RTCs involving unlicensed drivers were over-represented among male drivers, suspected impaired drivers, severe injuries, crashes occurring in higher speed limit areas, and in fair road conditions. Unlicensed drivers from families in a lower socioeconomic position showed increased relative risks for RTC in the range of 1.75 to 3.25. Those living in rural areas had an increased relative risk for a severe RTC of 3.29 (95% CI 2.47 - 4.39) compared to those living in metropolitan areas.

**Conclusions:**

At the time of the crash, young unlicensed drivers display more risky driving practices than their licensed counterparts. Just as licensed drivers, unlicensed young people from low socioeconomic positions are over-represented in the most severe injury crashes. Whether the mechanisms lying behind those similarities compare between these groups remains to be determined.

## Background

Young car drivers run a higher risk of road traffic crash (RTC) and road traffic injury (RTI) [1] not only because of their lack of experience but also because of their young age (and stage of development) and their greater propensity for adopting unsafe driving practices, including alcohol/drug consumption, high speed, night driving, and disregard for traffic regulations [2]. Also, just as the injury risks to children as unprotected road users are influenced by low socioeconomic position, the same is true for the crash involvement of young drivers, [3-10] whether this is a reflection of greater driving exposure or of differences in driving practice has been debated, but not well researched [5].

For their part, studies from the USA, Australia, Italy, New Zealand, and Great Britain on fatal RTCs [11-15] and on self-reported safe practices [16,17] indicate that unlicensed driving may be a concern - but a neglected issue - relative to young drivers. Driving unlicensed, in turn, might be biased to particular circumstances and settings [11]. A recent Swedish study indicates for instance an over-representation of unlicensed drivers in crashes involving young drivers characterized as single-vehicle crashes, alcohol/drug impairment, and night-time driving [18].

In Sweden, where the current study has been conducted, people must be 18 before they qualify for a driver's license and the process is relatively costly. Even then, less than a quarter gain a license during their first year of eligibility [18]. The age and socioeconomic distributions of young unlicensed drivers involved in a crash have yet to be determined. This paper aims to investigate RTCs among unlicensed and licensed young drivers with regard to characteristics and circumstances of the crash, and to examine the risk of a RTC among unlicensed young adults, including a comparison by age, socioeconomic position and living area.

## Methods

### Study population

This population-based cohort encompasses 1 616 621 individuals, born between 1977 and 1991, who were in the Swedish Population Register on 31 December 1997. Information regarding family socioeconomic position, level of population density, and RTCs was linked to the cohort. Linkage between Swedish registers is possible due to the unique personal identification number assigned to each resident in Sweden [19]. All linkages were made by the authorities who are responsible for keeping and maintaining the registers. The cohort was closed to immigration and followed with regard to RTCs during 1998 to 2004. A description of the cohort is presented in Table 1.

**Table 1:** Characteristics of the study population, (n = 1 616 621), percentage.

Characteristic	%
Sex	
Male	51.34
Female	48.66
	
Household socioeconomic position	
High/intermediate level salaried employees	37.95
Farmer	6.96
Self-employed	1.97
Skilled/unskilled workers	33.71
Assistant non-manual employees	12.77
Others	6.64
	
Receipt of social welfare benefits	
Yes	23.47
No	76.53
	
Urbanicity	
Metropolitan areas^1^	32.89
Large urban areas^2^	36.75
Medium-sized urban areas^3^	18.11
Small urban areas^4^	5.97
Rural areas^5^	6.29

^1 ^Metropolitan areas (Stockholm, Gothenburg and Malmö), more than >300 000 inhabitants

^2 ^Large urban areas, >90 000 inhabitants within 30 kilometres of city centre

^3 ^Medium-sized urban areas, 27 000- 90 000 inhabitants within 30 kilometres of city centre and >300 000 inhabitants within 100 kilometres of the same city centre

^4 ^Small urban areas, 27 000-90 000 inhabitants within 30 kilometres of city centre and <300 000 inhabitants within 100 kilometres of the same city centre

^5 ^Rural areas, <27 000 inhabitants within 30 kilometres of city centre

### Outcomes

The study was restricted to RTCs involving four-wheeled passenger vehicles (22 300 such RTCs were registered within the cohort during the study period) and thereafter to first-time car crash during follow-up (21 386 crashes).

Information on police-reported RTCs was derived from the Swedish National Road Administration Database from 1998 to 2004. Crash information is recorded by the police at the crash site and includes age and sex of the driver; suspicion of impaired driving due to alcohol/drugs; type and severity of injury to the driver and most serious injury to all others in the crash separately; driving conditions including speed restriction; weather and road conditions; time and urbanization level of crash site. Those data are updated to include deaths occurring within 30 days after the crash. It was not possible to obtain data on number of vehicles involved in the crash through the full study period due to changes in coding routines in 2003.

RTIs to drivers were classified into four categories: (1) no physical injury; (2) minor injuries not requiring hospital care; (3) serious injuries requiring hospital care; and (4) fatalities. The most serious injury outcomes to other persons involved were classified similarly, but RTCs with no physical injuries were included in the minor injuries category. Severe RTCs were defined as leading to serious injuries requiring hospital care or fatality among any of the persons involved.

### License status

License issue dates were gathered from the National Driver's License Register administered by the Swedish National Road Administration. The register contains information on license issue dates and vehicle endorsement. In Sweden, as mentioned above, people must be 18 years old before they can take their full driver's license. They may however start to learn at the age of 16 but they must have a learner's permit and be accompanied by a person with valid license while driving [18]. Study subjects without a date of issue of a full driver's license at the time of crash were defined as unlicensed drivers. Subjects were regarded as licensed drivers from the date the license was issued. Information regarding revoked licenses was not available.

### Socioeconomic position

Information on household socioeconomic position was gathered from the Population and Housing Census of 1990. Each parent's social position was defined according to a classification used by Statistics Sweden based on parent's occupation. Also, the family's weighted socioeconomic group was used based on the "dominance" principle developed by Erikson [20,21]. Each participant was allocated to one of the following six socioeconomic groups: Intermediate and high-level salaried employees; farmer (small-scale and medium-scale farmers); self-employed (self-employed without employees or small-scale entrepreneurs); assistant non-manual employees; manual workers (skilled and unskilled); and others (such as students, persons on sickness leave and disability pensions, and the long-term unemployed). In the analyses, young people from families with intermediate and high-level salaried employees were used as the reference group.

### Income, social welfare benefits, and parental car ownership

Information on family disposable income per unit of consumption (in 1998) and social welfare benefits (participants whose parents at some point in time during 1993 to 1997 received social welfare benefits) was obtained from Sweden's Total Enumeration Income Surveys. Information about parental car ownership (in 1998) was gathered from the Swedish Motor Vehicle Register.

### Urbanicity of living area

Urbanicity was defined according to the living areas of the subjects in 1998, and was divided into five categories based on population density and proximity to the city centre. Categories include metropolitan areas (>300 000, Stockholm, Gothenburg and Malmö), large urban areas (>90 000 within 30 kilometres of city centre), medium-sized urban areas (27 000- 90 000 within 30 kilometres of city centres and >300 000 within 100 kilometres of the same city centre), small urban areas (27,000-90,000 within 30 kilometers of city centre and <300,000 within 100 kilometres of the same city centre), and rural areas (<27 000 within 30 kilometres of city centre).

### Statistical analysis

#### Cumulative incidence

**Figure 1:**
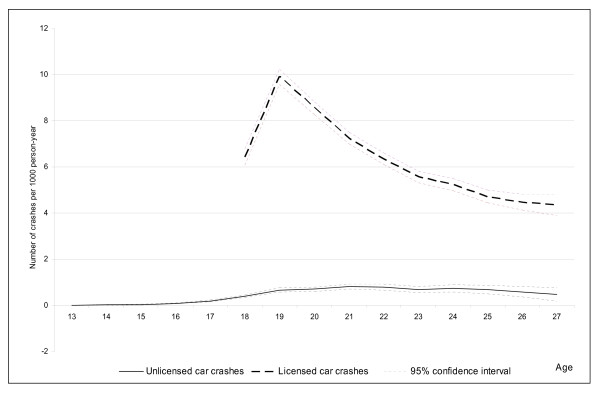
**Age-specific cumulative incidence of first car crash during 1998-2004 per 1000 person years, with 95% confidence intervals**.

The seven-year cumulative incidence of RTC per 1 000 person years with 95% confidence intervals (presented in Figure 1) was calculated as the ratio of the number of RTCs per year at each age by the person-time at risk. Person-time at risk for unlicensed drivers was calculated by age by adding up the time until the date of licensing. As there were very few unlicensed RTCs that occurred before the age of 13, Figure 1 presents the results of our compilations from 13 years and above. All individuals have been followed for 7 years and during these years some individuals changed from being unlicensed to licensed drivers, hence they contribute with person-time in the calculation of the cumulative incidence of RTC within the unlicensed group while being unlicensed and within the licensed group while being licensed.

#### Licensed vs. unlicensed RTCs

Table 2 compares the circumstances of RTCs occurring among unlicensed and licensed drivers respectively during the follow-up period, one variable at a time. Proportions by category of variables are reported and proportions among licensed and unlicensed drivers are compared using p-values for chi-square test.

**Table 2:** Percentage and Chi-square values with p-values of characteristics and circumstances of RTC involving a young driver stratified by license status, 1998 to 2004 (n = 21 386)

	Licensed n = 19864	Unlicensed n = 1522	Chisq value	Degrees of freedom	P-value for chisq
Sex					
Male	72.50	85.09	114.7	1	<.0001
Female	27.50	14.91			
					
Suspected impaired driving					
Yes	4.54	43.72	2571.0	1	<.0001
No	95.46	56.28			
					
Injury outcome for the young driver involved in the crash^1^					
Fatal	0.01	18.97	3842.2	3	<.0001
Severe injury	10.22	16.44			
Minor injury	57.19	45.41			
No injury	32.59	19.17			
					
Most serious injury outcome for other persons involved in the crash^2^					
Fatal	1.19	20.76	2215.8	2	<.0001
Severe injury	17.42	21.81			
Minor injury	81.39	57.42			
					
Speed restriction limit					
50 km/hour or less	45.65	41.98	6.9	2	0.0324
70 km/hour	26.94	27.92			
90 km/hour or more	27.41	30.10			
					
Time of day					
0600-1859	67.70	44.15	552.5	2	<.0001
1900-2259	18.25	18.97			
2300-0559	14.05	36.88			
					
Light conditions					
Daylight	57.98	41.16	161.2	2	<.0001
Darkness	33.83	47.54			
Dusk/dawn	8.19	11.30			
					
Weather conditions					
Dry (fair)	79.82	82.27	15.1	4	0.0046
Haze	2.72	3.56			
Rain	11.78	10.20			
Sleet	1.82	1.51			
Snow	3.85	2.46			
					
Road surface conditions					
Dry	54.11	58.91	25.7	4	<.0001
Wet/damp	29.24	29.08			
Thick ice/packed snow	3.44	2.46			
Thin ice	7.68	6.21			
Light snow/snow slush	5.54	3.34			
					
Urbanicity of crash site					
Urban area	52.61	46.98	17.8	1	<.0001
Rural area	47.39	53.02			

^1 ^Injury outcomes for the young driver were classified into four categories: (1) no physical injury; (2) minor injury not requiring hospital care; (3) serious injury requiring hospital care; and (4) fatality.

^2 ^The most serious injury to other persons involved in the crash were classified thus: (1) no or minor injury not requiring hospital care; (2) serious injury requiring hospital care; and (3) fatality.

#### Hazard ratios among unlicensed drivers

To study the effect of socioeconomic positions and level of urbanicity on the risk of first-time RTC among unlicensed young people, we calculated hazard ratios with corresponding 95% confidence intervals (CIs) as measures of relative risks (RR) using Cox regression. Each cohort member contributed with person-time from the entry date (31 December 1997) until the date of the first RTC, death, emigration from Sweden, date of issued license or to the end of 2004, whichever occurred first. The results of the analyses are presented (see Table 3) for all crashes and for severe RTCs. Relative risks are presented as crude and as adjusted for sex and age as a continuous variable (by stratification allowing the baseline hazard function to vary for the different age-cohorts). The crude and adjusted analyses were based on the same number of individuals. Further adjustments for income (based on household disposable income 1998) and car ownership (based on parental registered car ownership in 1998) were tested (data not shown). Study subjects with missing values on exposures, varying from 0.004% for living area to 4.6% for household socioeconomic position, were excluded in the analyses and the number of persons included in the analyses varied between 1 477 743 and 1 404 703.

**Table 3:** Relative risks with 95% confidence intervals of RTC of unlicensed drivers stratified by injury severity, 1998-2004.

	All RTCs, n = 1522		Severe^1 ^RTCs, n = 648	
	Crude	Adjusted^2^	Crude	Adjusted^2^
Sex	n = 147743		n = 1476869	
Male	5.74 (4.98-6.61)		6.57 (5.24-8.25)	
Female	1.0		1.0	
				
Household socioeconomic position	n = 1405498	n = 1405498	n = 1404703	n = 1404703
High/intermediate level salaried employees	1.0	1.0	1.0	1.0
Farmer	2.11 (1.70-2.63)	2.08 (1.67-2.58)	2.43 (1.75-3.37)	2.40 (1.73-3.33)
Self-employed	1.43 (0.89-2.30)	2.04 (1.27-3.29)	2.73 (1.57-4.75)	4.18 (2.40-7.28)
Assistant non-manual employees	1.62 (1.34-1.96)	1.75 (1.45-2.11)	1.85 (1.39-2.46)	2.00 (1.51-2.66)
Manual workers	2.02 (1.76-2.32)	2.28 (1.99-2.62)	2.30 (1.86-2.84)	2.60 (2.10-3.22)
Others	3.49 (2.93-4.15)	3.25 (2.73-3.88)	2.95 (2.21-3.94)	2.72 (2.04-3.63)
				
Receipt of welfare benefits	n = 1477743	n = 1477743	n = 1476869	n = 1476869
Yes	2.97 (2.68-3.28)	2.21 (1.99-2.44)	2.52 (2.16-2.94)	1.81 (1.55-2.12)
No	1.0	1.0	1.0	1.0
				
Urbanicity^3^	n = 1472083	n = 1472083	n = 1471215	n = 1471215
Metropolitan areas	1.0	1.0	1.0	1.0
Large urban areas	0.96 0.85-1.08	1.14 1.01-1.28	1.24 (1.02-1.50)	1.50 (1.24-1.83)
Medium-sized urban areas	0.91 0.78-1.06	1.20 1.03-1.40	1.30 (1.03-1.64)	1.76 (1.39-2.23)
Small urban areas	0.96 0.76-1.40	1.30 1.03-1.64	1.41 (1.00-1.98)	1.97 (1.40-2.77)
Rural areas	1.14 0.92-1.40	1.71 1.38-2.11	2.09 (1.57-2.79)	3.29 (2.47-4.39)

^1 ^Severe RTCs were defined as those leading to serious injuries, requiring hospital care or fatal for any persons involved in the RTC.

^2 ^Adjusted for sex and age.

^3 ^For definitions please see Table 1.

The study was submitted for ethical review to and approved by The Regional Ethical Review Board in Stockholm.

## Results

Figure 1 shows that RTCs among unlicensed drivers are not a phenomenon restricted to under-age drivers but persist beyond the age of licensing. RTCs among unlicensed drivers younger than 13 were extremely unusual. The extent of RTCs is markedly increased at the age of 18 years for both licensed and unlicensed young people.

### Circumstances of licensed and unlicensed crashes

There were several statistically significant differences in the crash circumstances of licensed and unlicensed drivers (see Table 2). The following proportions were significantly higher among unlicensed drivers: male drivers, suspected impaired drivers, injury severity for both the driver and other persons involved in the RTC, speed restriction limit (>70 km/hr), time of crash (23:00-05:59), light conditions (darkness and dawn), weather (dry and haze), and road surface conditions (dry), and traffic environment (rural). A sub-analysis of the data from 1998-2002 showed that the proportion of single crashes was twice as high among unlicensed drivers (73.3%) (data not shown).

Table 3 shows nearly a six-fold increase for RTC among males (RR = 5.74, 95% CI 4.98-6.61). Subjects in all socioeconomic groups showed increased risk for RTCs as an unlicensed driver compared to the reference group of subjects in families with high/intermediate salaried employees, ranging from RR = 1.75 (95% CI 1.45-2.11) for "assistant non-manual employees" to 3.25 (95% CI 2.73-3.88) for "others". Young people from families with a history of receiving social welfare benefits ran twice as high a risk for RTC (RR = 2.21, 95% CI 1.99-2.44) compared to young people from families without such a history. Living outside metropolitan areas also resulted in elevated relative risks, with the highest risk estimate in rural areas (RR = 1.71 95% CI 1.38-2.11). Restricting the analyses to severe RTC showed the same overall pattern of increased relative risks. However, young people with self-employed parents had a much higher risk estimate for severe RTCs, RR = 4.18 (95% CI 2.40-7.38), compared to RR = 2.04, (95% CI 1.27-3.29) for all RTCs. Further, the risk for a severe RTC was much higher in rural areas, (RR = 3.29 (95% CI 2.47-4.39).

Adjustment for family disposable income and car ownership only slightly changed the effect estimates (data not shown).

## Discussion

### Main findings

Among young Swedish drivers, injury in an RTC as an unlicensed driver occurs not only prior to the age of licensing eligibility but also thereafter, and at a rather stable rate until the age of 27. The study is restricted to unlicensed drivers and does not consider those driving while having a permit suspended or revoked. The rationale for this focus is that not being licensed at all is more a reflection of people not engaging in - or completing - the driver training process even after several years of becoming eligible. This is intriguing, seldom reported, and deserves attention. Further, the RTCs involving unlicensed drivers differ from those involving their licensed counterparts with regard to both crash circumstances and injury severity. As more risky driving practices have been associated with severe and fatal RTCs, it is not surprising that crash severity is higher among unlicensed drivers [18,22]. Whether those risks are specific to crash circumstances or reflect a trait more typical of unlicensed drivers in general remains to be determined. Yet, self-reported studies of young people also indicate that unlicensed driving tends to occur as "joy-riding" and without purpose, typically at night and weekends, and while under the influence of alcohol [16,17].

In addition, the study shows that being involved in a severe crash as an unlicensed driver is more common among young people who are not from families of the highest socioeconomic position and who live outside metropolitan areas. The former finding echoes an earlier Swedish study on young licensed drivers,[7-10] but we still lack information as to whether the mechanisms are comparable. Is driving unlicensed more prevalent among young people not from the highest socioeconomic position (e.g. licensing barriers) [23]? Are they more prone to risk-taking (e.g. crash likelihood differences) [21,24]? Or are the consequences of the crashes they are involved in more severe (e.g., protection differences) [25]? All three hypotheses are plausible.

The licensing process provides the driver with the minimum skills and experience needed to operate a motor vehicle safely. There are indications that the opportunity to prepare for a license are unequal as a result of less access to vehicles and poorer driving opportunities for young people from families of lower socioeconomic position [10,23]. The fact that unlicensed drivers are twice as likely to be involved in a single-vehicle crash in which they lost control of the vehicle suggests their lack of formal driving preparation is a factor.

Unlicensed drivers from self-employed families had a higher risk estimate for severe injury than has been reported in other studies [7,9]. We found that they are more likely to be licensed earlier indicating a need to be mobile perhaps as part of a family business [10]. It seems as though - and not surprisingly -younger-age driving combined with increased exposure increases the risk.

Finally, unlicensed drivers living in rural areas, compared to those living in metropolitan areas, showed a much higher risk for RTCs with severe injury outcome. The imbalance is possibly due to driving exposure because of, inter alia, the necessity of travelling greater distances in higher speed areas, and the lack of commuting alternatives such as public transport [26]. Even factors like inadequate pre-hospital care after a crash may influence the unequal geographical distribution of the most severe injuries in rural areas [27,28]. Whether the latter applies to the distribution of unlicensed driving in Sweden is not known.

This study contributes important and new information regarding RTCs involving unlicensed drivers. The combination of linking multiple databases containing population, socioeconomic, and crash data provides important insight into the social stratification of RTCs and RTIs. Our study population consists of a large cohort from the Swedish Population and Housing Census Database that is fully representative of the Swedish population and continually updated. License status was gathered from the National Driver's License Register and covers all licenses issued in Sweden. One limitation is that we did not have information regarding revoked licenses, implying possible misclassification of some young people as licensed, and a slight underestimation of the incidence of unlicensed RTCs.

The Swedish National Road Administration Register covers all police-reported RTCs during the seven-year follow-up. It is, however, well known that police crash reports do not give an exhaustive picture of the number of RTCs, especially underestimating RTCs that do not give rise to serious injuries. The police may pay closer attention to specific persons in a crash, especially if that person is a young driver suspected to be under the influence of alcohol/drug. Accuracy and completeness of crash data are also restricted to the reporting and subjective assessment of the police at the scene [29].

In the cohort analyses, all exposures were assessed through registers implying reduced risk for information bias. However, exposure was assessed at the time of inclusion. The young people were classified according to the socioeconomic position of their parents. For the early birth cohorts, aged 20 at inclusion, this might be misleading as during the seven-year follow-up period, they may establish their own socioeconomic position independent of the family's. Upward social mobility for young people in this study would lead to an underestimation of the relative risk among lower socioeconomic groups.

Confounding in population-based studies of road traffic safety is difficult to control even under the best of circumstances. Our estimation of person-years at risk, based on time of license status, does not take into account the extent to which young people from different socioeconomic groups and levels of urbanicity have similar driving profiles in terms of conditions, types of vehicles, and distances driven. Included in the rate are licensed and unlicensed drivers who may have zero driving exposure. The commitment to road traffic safety in Sweden is supported by culturally and socially defined norms of acceptance and compliance with traffic safety measures that possibly contribute to decreased exposure among some socioeconomic positions [30].

Young people's access to a vehicle is highly dependent on the availability of a family car and household disposable income. However, car ownership is high in Sweden with 86% of the subjects- families having a registered car during 1998. Adjusting our analyses and taking into account household disposable income and car ownership did not alter our conclusions.

The results can be generalized to other settings in high-income countries with similar socioeconomic differences and motor traffic systems. Even though fatal crashes for both licensed and unlicensed young drivers are relatively rare in Sweden, the social patterning and area distribution of RTCs among unlicensed drivers may be similar in other countries. Access to population-based socioeconomic and crash data in Sweden is important in understanding the mechanisms of unlicensed driving.

## Conclusions

Unlicensed driving is an eligibility-to-licensing question as a majority of crash-involved unlicensed drivers were old enough to get a driving permit. This, in turn, raises questions about how accessible licensing is in Sweden and the determinants of young peoples- decisions to get a permit or to drive unlicensed. This is of importance as this study reveals that the circumstances surrounding crashes involving young unlicensed drivers imply significantly more risky driving behaviors and lead to severe injury. Also, as is the case for young licensed drivers, lower socioeconomic position is associated with higher crash involvement. For its part, the excess risk of severe injury in RTCs involving unlicensed drivers living in areas with less population density in Sweden is a new finding.

## Competing interests

The authors declare that they have no competing interests.

## Authors' contributions

CH contributed to the conception and interpretation of the data, drafted the manuscript and was responsible for the overall content under the supervision of LL. LL conceived the study idea and contributed to the conception, interpretation of data, drafting of the manuscript and helped to supervise CH. MH contributed to the conception and design of the study, acquisition of data, interpretation of data and drafting of the manuscript. JM contributed to the conception and design of the study, made the statistical analyses and interpretation of data, and helped to draft the manuscript. All authors have read and revised the manuscript for important intellectual content, and approved the last version of the manuscript.

## Pre-publication history

The pre-publication history for this paper can be accessed here:

http://www.biomedcentral.com/1471-2458/10/14/prepub
